# German hospital capacities for prolonged mechanical ventilator weaning in neurorehabilitation – results of a representative survey

**DOI:** 10.1186/s42466-020-00065-1

**Published:** 2020-07-01

**Authors:** Thomas Platz, Andreas Bender, Christian Dohle, Anna Gorsler, Stefan Knecht, Joachim Liepert, Thomas Mokrusch, Michael Sailer

**Affiliations:** 1 Presidium of the German Neurorehabilitation Society (Deutsche Gesellschaft für Neurorehabilitation, DGNR e.V.), Rheinbach, Germany; 2 BDH-Klinik Greifswald, Neurorehabilitation . Ventilation and Intensive Care . Spinal Cord Injury Unit, Karl-Liebknecht-Ring 26a, 17491 Greifswald, Germany; 3 Neurorehabilitation Research Group, Universitätsmedizin Greifswald, Greifswald, Germany

**Keywords:** Neurorehabilitation, Weaning, Neuro-disabilities, Hospital capacity

## Abstract

A brief survey among members of the German Neurorehabilitation Society aimed to document the hospital capacities (“beds”) for prolonged weaning from a mechanical ventilator for patients with neuro-disabilities that require simultaneous multi-professional neurorehabilitation treatment. Sixty-eight institutions declared to have capacities with a broad distribution across Germany and its federal states. Overall, 1094 “beds” for prolonged weaning (and neurorehabilitation) were reported, 871 together with further information regarding their identification and hence regional location. These units had on average 16.1 beds for prolonged weaning (95% confidence interval 12.6 to 19.6) with a range from 2 to 68 beds per organization. The data indicate substantial capacities for the combined prolonged weaning and neurorehabilitation treatment in Germany. For most “beds” included in this analysis a basic validation was possible. While a reasonable coverage of these specialized service capacities by the survey is likely, the number reported could still be biased by underreporting by non-response. Both the broad variation of number of “beds” for prolonged weaning per unit and their unequal geographical distribution across federal states (per capita rate) warrant a more refined follow-up survey that will provide insights into reasons for the observed pattern of variation for these specialized hospital capacities.

## Introduction

Weaning is the medical process of withdrawing ventilator support. Prolonged weaning describes a situation of initial weaning failure, i.e. when more than three spontaneous breathing trials (SBT) or 7 days from the first SBT are required, and hence prolonged weaning care [1]. In specialized pulmonologic weaning centers, about 50% of all patients with initial weaning failure can be liberated from mechanical ventilation [2].

However, a substantial subset of patients in need for prolonged weaning treatment is also affected by neuro-disabilities and requires the combination of prolonged weaning treatment and multi-professional neurorehabilitation to address their various needs for improving both their health, body functions, and autonomy with activities of daily living [5]. In a German cohort, 26% of 754 patients admitted for “early neurorehabilitation” were on mechanical ventilation commencing their neurological rehabilitation; their weaning rate from mechanical ventilation was 65% during their stay [4].

While there is a considerable need for such a specialized combined service with proven effectiveness, there is a lack of knowledge about such hospital capacities that are currently available in Germany.

The German Neurorehabilitation Society (Deutsche Gesellschaft für Neurorehabilitation, DGNR e.V.) conducted a survey among its members to document hospital capacities (“beds”) for prolonged weaning for patients with neuro-disabilities that require simultaneous multi-professional neurorehabilitation treatment (“neuro-weaning beds”).

## Methods

By email invitation end of December 2019 and a repeated invitation at the beginning of January 2020, 381 members of the DGNR were invited to participate in a short online survey. They were asked to answer two questions hosted on the platform invote.de (provided by Netzmanufaktur GmbH, Theaterstraße 4, 01067 Dresden):

1. Number of “neuro-weaning beds” in their hospital
2. Combined question to indicate number of “neuro-weaning beds”, organizational background (acute care hospital versus rehabilitation facility), name of the hospital, and name of head of department.

By repeating the question for number of beds and by asking for more detailed (identifying) information, the validation of entries was sought to be promoted. In addition, society members were encouraged to make sure within their hospital by contact with their head of department that data entry was provided only once per hospital to prevent reporting in duplicate.

All entries were screened for validity. Based on hospital name and head of department name the location of each unit within one of 16 German Federal states was coded.

The number of “neuro-weaning beds” per federal state was divided by population statistics for that state as published by the German Federal Agency for Statistics [7] to obtain the rate of “neuro-weaning beds” per 1.000.000 inhabitants.

Descriptive statistics were generated using the software package SAS.

## Results

Sixty-nine of a total of 123 survey respondents indicated that their hospital provides capacities for combined prolonged weaning and neurorehabilitation (“neuro-weaning beds”).

One entry was regarded as “invalid entry”: The entry stated “135” (beds) without further information and was not used for the descriptive statistics (as stated below).

The 68 remaining units had a total of 1094 “neuro-weaning beds”, on average 16.1 beds (95% confidence interval 12.6 to 19.6) with a range from 2 to 68 beds per organization. Given a total population of 82.792 thousand inhabitants in Germany [7] this statistic would imply a capacity of 13.2 “neuro-weaning beds” per 10^6^ inhabitants.

As a sensitivity analysis we further analyzed the subset of data from units that gave more detailed (identifying) information (*n* = 57). Collectively these units reported 871 “neuro-weaning beds” with on average 15.3 beds for prolonged weaning (95% confidence interval 11.7 to 18.8) and a range from 2 to 68 beds per organization. Three of these units were specialized for health care in children and adolescents (22 beds), two units for people with spinal cord injury (12 beds).

The (subset of) units that reported “neuro-weaning beds” together with their identification served as basis to describe their distribution across federal states in Germany (see Table 1).

**Table 1:** Distribution of reported hospital capacities for combined prolonged weaning and neurorehabilitation across federal states in Germany

Federal state	Number of units	Number of beds	Beds per 10^6^ inhabitants
Baden-Württemberg	8	80	7.3
Bayern	8	138	10.6
Berlin	4	37	10.2
Brandenburg	5	85	33.9
Bremen	0		
Hamburg	3	88	48.1
Hessen	5	114	18.3
Mecklenburg-Vorpommern	3	75	46.6
Niedersachsen	8	85	10.7
Nordrhein-Westfalen	4	46	2.6
Rheinland-Pfalz	2	21	5.2
Saarland	0		
Sachsen-Anhalt	1	18	18.1
Sachsen	2	26	6.4
Schleswig-Holstein	3	53	18.3
Thüringen	1	5	2.3

## Discussion

This representative survey indicated substantial hospital capacities for combined prolonged weaning and neurorehabilitation with a total of 1094 “neuro-weaning beds” in Germany. For 871 of these “neuro-weaning beds” identifying information was available supporting the survey’s validity.

As with any voluntary survey, there is a relevant risk of underreporting. Thus, the true number of “neuro-weaning beds” in Germany is likely to be higher than the one reported here.

The number of “neuro-weaning beds” within individual reporting hospitals varies considerably (from 2 to 68 beds) with an average of 16 beds and a 95% confidence interval ranging from 12 to 19. This indicates both a central tendency for organizational size and substantial differences in organizational settings.

The analysis of the geographical distribution across federal states of Germany was based on the 80% of “neuro-weaning beds” reported with identifying information. Hence, this analysis suffers from an “incomplete data” bias and absolute numbers should be interpreted with great caution. The data nevertheless points to a huge variability of population-based density of “neuro-weaning beds” (per 10^6^ inhabitants for German federal states) in Germany.

## Conclusions

The survey generated a crude estimate of hospital capacities (“beds”) for prolonged weaning from a mechanical ventilator for patients with neuro-disabilities that require simultaneous multi-professional neurorehabilitation treatment. The substantial variability in size of units and their geographical distribution warrants a more refined follow-up survey to learn about the setting and organizational structures of such units before further conclusions can be drawn. However, this survey already confirms the high relevance of these “neuro-weaning” capacities for the recovery from breathing failure and hence the avoidance of long-term intensive home care. The number of people in need for invasive long-term ventilation in Germany dramatically increased over the past 15 years to an estimate of currently 20.000 patients, implying additional health care costs of around 4 billion Euros per year [3]. It is estimated that approximately 10.000 patients with neuro-disabilities in need for weaning from mechanical ventilation can be taken care of each year with the capacity of 1094 “neuro-weaning beds” of this survey. Given a success rate of 65 to 75% [4, 6], they collectively might prevent an estimated 7000 new cases of long-term ventilation per year, let alone the other neurorehabilitation achievements in terms of disability reduction and re-gaining autonomy with activities of daily living.

## Data Availability

The datasets generated during this survey are not publicly available, since no consent to share the hospital-based information has been obtained. Confidential inspection of the data is possible at the site of the corresponding author on reasonable request.

## Abbreviations

DGNR: Deutsche Gesellschaft für Neurorehabilitation e.V.
SBT: Spontaneous breathing trial
